# Semi-automated classification of colonial *Microcystis* by FlowCAM imaging flow cytometry in mesocosm experiment reveals high heterogeneity during seasonal bloom

**DOI:** 10.1038/s41598-021-88661-2

**Published:** 2021-04-30

**Authors:** Yersultan Mirasbekov, Adina Zhumakhanova, Almira Zhantuyakova, Kuanysh Sarkytbayev, Dmitry V. Malashenkov, Assel Baishulakova, Veronika Dashkova, Thomas A. Davidson, Ivan A. Vorobjev, Erik Jeppesen, Natasha S. Barteneva

**Affiliations:** 1 School of Sciences and Humanities, Nazarbayev University, Nur-Sultan, 010000 Kazakhstan; 2 National Laboratory Astana, Nur-Sultan, 010000 Kazakhstan; 3 Department of General Ecology and Hydrobiology, Lomonosov Moscow State University, 119991 Moscow, Russian Federation; 4 School of Engineering and Digital Sciences, Nazarbayev University, Nur-Sultan, 010000 Kazakhstan; 5 Department of Bioscience, Aarhus University, 8600 Silkeborg, Denmark; 6 Institute of Marine Sciences, Middle East Technical University, Mersin, 33731 Turkey; 7 Limnology Laboratory, Department of Biological Sciences and Centre for Ecosystem Research and Implementation, Middle East Technical University, Ankara, 06800 Turkey; 8 Sino-Danish Centre for Education and Research, Beijing, 100049 China; 9 The Environmental Research and Efficiency Cluster (EREC), Nazarbayev University, Nur-Sultan, 010000 Kazakhstan; 10 Present Address: University of British Columbia, Vancouver, Canada

**Keywords:** Biological techniques, Limnology

## Abstract

A machine learning approach was employed to detect and quantify *Microcystis* colonial morphospecies using FlowCAM-based imaging flow cytometry. The system was trained and tested using samples from a long-term mesocosm experiment (LMWE, Central Jutland, Denmark). The statistical validation of the classification approaches was performed using Hellinger distances, Bray–Curtis dissimilarity, and Kullback–Leibler divergence. The semi-automatic classification based on well-balanced training sets from *Microcystis* seasonal bloom provided a high level of intergeneric accuracy (96–100%) but relatively low intrageneric accuracy (67–78%). Our results provide a proof-of-concept of how machine learning approaches can be applied to analyze the colonial microalgae. This approach allowed to evaluate *Microcystis* seasonal bloom in individual mesocosms with high level of temporal and spatial resolution. The observation that some *Microcystis* morphotypes completely disappeared and re-appeared along the mesocosm experiment timeline supports the hypothesis of the main transition pathways of colonial *Microcystis* morphoforms. We demonstrated that significant changes in the training sets with colonial images required for accurate classification of *Microcystis* spp. from time points differed by only two weeks due to *Microcystis* high phenotypic heterogeneity during the bloom. We conclude that automatic methods not only allow a performance level of human taxonomist, and thus be a valuable time-saving tool in the routine-like identification of colonial phytoplankton taxa, but also can be applied to increase temporal and spatial resolution of the study.

## Introduction

Studying plankton organisms is critical to assess the health of ocean and freshwater ecosystems. Over the past decade, a combination of image analysis technologies and machine learning algorithms has been applied to characterize zooplankton^1–5^ and phytoplankton organisms^6–13^. Light microscopy is still considered the golden standard technique providing high-resolution plankton images for qualitative and quantitative assessment. However, microscopy is a time-consuming approach that requires a high level of taxonomic skills and can result in human-based misclassification and underestimation of rare species^14–16^. Moreover, microscopy identification of plankton is limited by an increased sample variability and diversity of the spatial orientation of the plankton organisms in the imaging plane, presence of organic matter particles in the water samples, and decay. There is, therefore, a high demand for automating the process of classification to enable high-throughput data processing. In the last decades imaging cytometers such as FlowCytobot^6^, FlowCAM^5,8,12,13^, and Imagestream X Mark II^9^ have been used to improve and speed up phytoplankton image acquisition. The FlowCAM instrument has become a valuable tool in marine and freshwater plankton studies because it enables researchers to classify, count, and monitor different plankton organisms^17,18^ in the preferred detection size range of 20–300 μm^19^. Image analysis and classification of large image datasets are primarily sensitive to high variations, manual misclassifications, and biased interpretations^20^. However, so far, phytoplankton analysis with imaging cytometers has mainly been limited to the genus level and not included differentiation of colonial morphospecies^5–13^.

*Microcystis* spp. is a dominant cyanobacterial genus appearing in all regions of the world. *Microcystis* can form toxic blooms whose occurrence is expanding; thus, more than 100 countries worldwide have documented such toxic blooms in freshwater lakes and streams^21^. Toxic strains of *Microcystis* produce hepatotoxins and neurotoxins^22,23^ that constitute a serious threat to human health by contaminating drinking water resources. The toxins of *Microcystis* spp. have harmful effects on different trophic levels in an aquatic food web, such as phytoplankton, zooplankton, fish, and mollusks^24–28^. Depending on the prevailing environmental conditions, *Microcystis* tend to form colonial structures covered by a thick polysaccharide sheath (mucilage)^29^. Colony formation by *Microcystis* can be induced by low temperatures, low light intensity, high lead ion concentrations, and the presence of other cyanobacterial species. According to Zheng et al.^30^, under laboratory conditions *Microcystis* spp. occur only as single or paired cells, preventing replication and study *Microcystis* spp. bloom formation. Morphospecies (or morphoforms) were identified in the *Microcystis* genus^31^, and their physiology, growth, and toxicity vary greatly^32^. Seasonal dynamics and increasing occurrence of water-bloom forming *Microcystis* is of great concern for the ecosystem due to the potential production of potentially toxic microcystins^33^, and *M. aeruginosa* is considered to be a major toxic morphospecies. *Microcystis* spp. occurs in the freshwater bodies mainly in a colonial form^34^, and their bloom dynamics were monitored by different research groups. Thus, in China, *Microcystis* blooms development and sustainment were studied in many lakes, including large Taihu and Dianchi lakes^25,35,36^. Thus, Otten and Paerl^36^ studied by genotyping the single colonies of four different morphoforms of *Microcystis* spp. that comprised seasonal blooms in Lake Taihu, and reported that one morphospecies was genetically unique (*M. wesenbergii*) and three (*Microcystis aeruginosa*, *Microcystis flos-aquae*, and *Microcystis ichthyoblabe*) were genetically indistinguishable (96.4% identity of 16S–23S ITS sequences). Ishikawa et al.^37^ examined *M. aeruginosa* and *M. wesenbergii* colonies in the Lake Biwa, Yamamoto, and Nakahara^38^ investigated *Microcystis* spp. in Hirosawa-no-ike Pond in Japan. Kurmayer and co-authors, Via-Ordorica and others studied *Microcystis* colonies in European freshwater bodies^39,40^, and Alvarez and co-authors in Uruguay^41^.

We used a unique LMWE mesocosm experiment (Aarhus University, Denmark)^42^ that, in contrast to the limited laboratory conditions, provides a dynamic system for the study of colonial phytoplankton. Previously, in 2018 we attempted classification of morphospecies in a study of the seasonal dynamics of different *Microcystis* spp.^43^. Five different *Microcystis* morphospecies (*M. aeruginosa, M. novacekii, M. smithii, M. wesenbergii*, and *M. ichthyoblabe*) were also detected and identified during the 2019 season. This study aimed to develop and validate a semi-quantitative machine learning algorithm for differentiation of *Microcystis* colonies intragenerically and from other phytoplankton colonial phytoplankton taxa (*Micractinium* genus) as well as from unicellular phytoplankton (*Cryptomonas* spp.). We distinguished five morphological colonial forms of *Microcystis* and found that the proposed intergeneric classification showed higher performance using minimized filter sets, whereas intrageneric differentiation had lower accuracy using high complexity filter sets*. *These semi-automated imaging cytometry-based classification results are comparable with the traditional human-based level of classification. We attribute the variations in intrageneric colonial analysis accuracy to the high heterogeneity of *Microcystis* spp. in the seasonal *Microcystis* bloom. Moreover, our observation that some *Microcystis* morphotypes completely disappeared and re-appeared along the mesocosm experiment timeline supports the hypothesis of the main transition pathways of colonial *Microcystis* morphoforms.

## Materials and methods

### Mesocosm experimental setup

**Figure 1:**
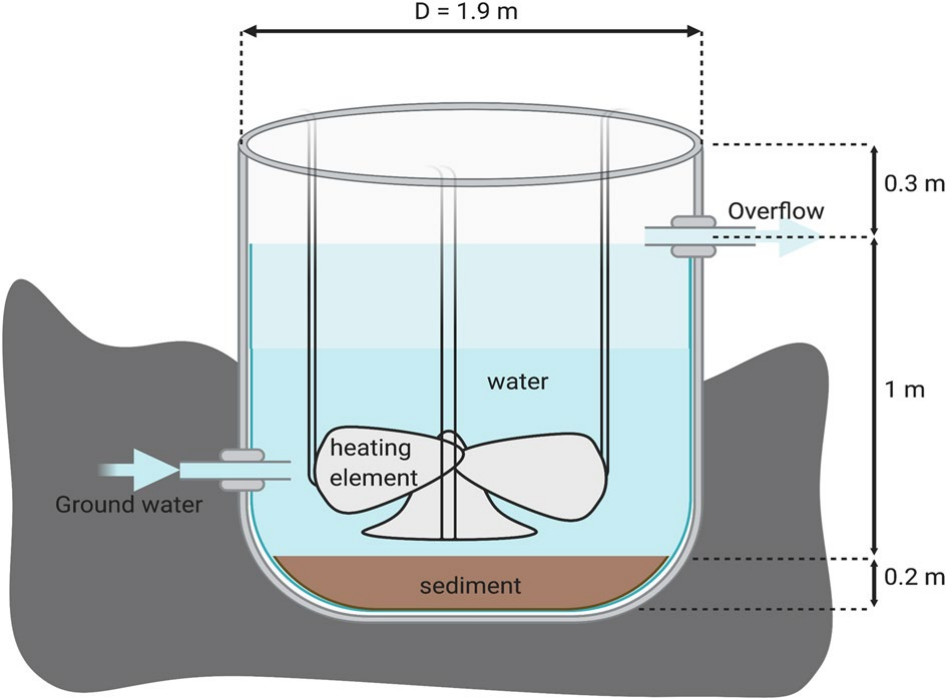
An image of one of the 24 flow-through tanks in the LMWE experiment run at the experimental facility belonging to Aarhus University, Denmark (modified from^42^). The collection tank was placed at right side (not included). This image was created with BioRender (https://biorender.com/).

We collected phytoplankton samples from the AQUACOSM Lake Mesocosm Warming Experiment (AQUACOSM LMWE experiment) in the experimental facility of Aarhus University in Central Jutland, Denmark (56°140 N, 9°310 E) to study different phytoplankton genera with a FlowCAM imaging cytometer. The mesocosm facility consists of 24 artificially mixed flow-through mesocosms that were established in August 2003. The factorial experimental set-up combines three temperature scenarios and two nutrient levels, all in four replicates (detailed description of experimental design and set-up can be found in Liboriussen et al.^42^). Overall, the LMWE experiment includes six different types of tanks with low and high nutrient levels that are each divided into three sub-types (depending on the temperature of the water: unheated, heated according to IPCC climate scenario A2, and eight heated according to A2 + 50%)—all with three replicates. In the present study, we collected seasonal (from May 23, 2019, to September 17, 2019) samples on 13 dates in the 12 high nutrient tanks, in a total of 168 samples. The tanks are named A1–3, D1–3, F1–3, and G1–3. A schematic representation of a mesocosm is shown in Fig. 1. The samples were preserved with glutaraldehyde solution (Sigma-Aldrich, USA) at a final concentration of 1% and analyzed using a FlowCAM imaging cytometer (Yokogawa Fluid Imaging Inc., USA).

### Instrumentation

We used a benchtop FlowCAM imaging cytometer equipped with VisualSpreadsheet software (Yokagawa Fluid Imaging, USA). Samples were recorded in autoimage mode using combinations of 10 × objective (NA = 0.3; resolution 1 pixel equals to 0.554 µm)/100 µm flow cell and/or 20 × objective/50 µm flow cell for identification, classification, and quantification. Identification and quantification of phytoplankton cells by light microscopy were performed under Leica DM500 (Leica Microsystems, Germany) equipped with phase contrast and series of objectives.

### Phytoplankton morphological classification

**Figure 2:**
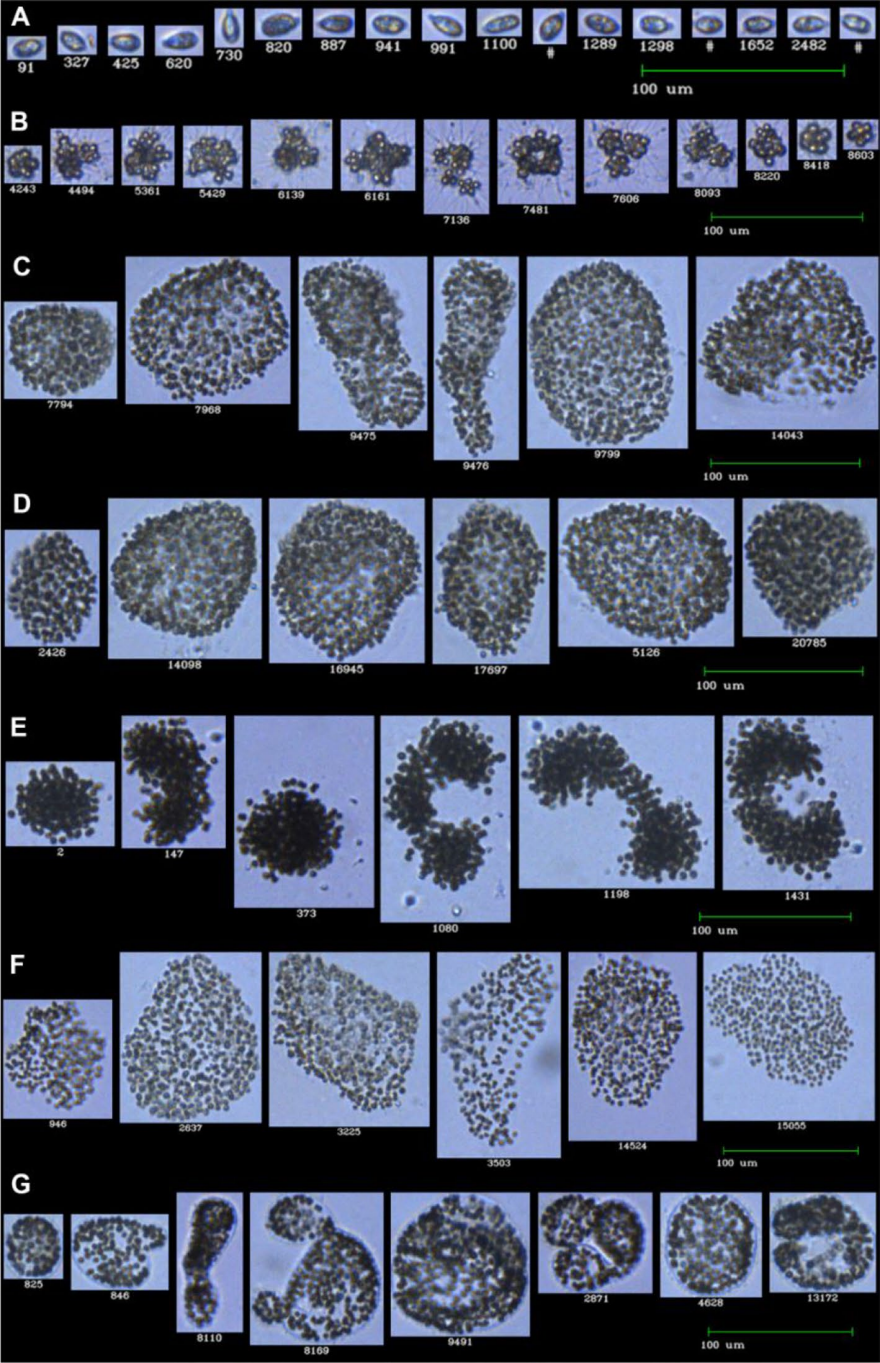
FlowCAM imaging flow cytometry of phytoplankton from the LMWE 2019 experiment. (**A**) *Cryptomonas *sp.; (**B**) *Micractinium*; (**C**) *Microcystis aeruginosa*; (**D**) *M. ichthyoblabe*; (**E**) *M. novacekii*; (**F**) *M. smithii*; (**G**) *M. wesenbergii.*

As stated before, we elucidated semi-automated classification for morphological analysis between a genus-level and colonial morphospecies dataset. Therefore, phytoplankton classification in this study was focused on on *Cryptomonas* sp*.*, *Micractinium*, and *Microcystis* morphospecies. *Microcystis* spp.was divided into five morphospecies, namely *M. aeruginosa*, *M. ichthyoblabe*, *M. novacekii*, *M. smithii*, and *M. wesenbergii*; and used this morphological difference between colonial morphospecies for intrageneric (within genus) classification. Training sets were developed with an expert taxonomist's participation (with > 10 years of experience). On the images, *Cryptomonas* sp*.* (hereafter *Cryptomonas*) were defined as brown-green cells with two flagella^44^. Examples from FlowCAM imaging are given in Fig. 2A. For *Micractinium*, spines/bristles were used for the identification, as illustrated in Fig. 2B. Five major morphospecies of *Microcystis *spp. were separated into classes of *M. novacekii, M. ichthyoblabe, M. smithii, M. aeruginosa*, and *M. wesenbergii* (see Fig. 2C–G). During a seasonal *Microcystis* bloom, some of the images show colony remnants with few or no cells. These images were assigned to the class “*Membrane*”. Small and dispersed non-colonial forms of *Microcystis* spp. classified as “*Undefined*”. The two latter classes were not used in the training sets in order to ensure clear separation between the five colonial morphospecies.

### Preparation of training set and dataset

Different mesocosm samples were mixed to achieve an optimal number of representatives from all three to examine intergeneric classification between colonial forms of *Microcystis, Micractinium, *and single cells of *Cryptomonas* genera. It is important to note that *Microcystis novacekii* was used in the training set as the only representative of the genus *Microcystis*. None of the tanks were found to contain all three representatives in the high image counts. Using the preliminary abundance analysis, four samples (D1_17/09/2019, D1_22/08/2019, G3_11/06/2019, G3_17/06/2019) were mixed in equal volumes, with a final volume of 10 mL. Then, bright-field images were recorded applying FlowCAM imaging flow cytometry in autoimage mode. Each sample was passed through a 100 μm filter and then recorded with a 10 × magnification objective (we observed only a few large colonies with a light microscope in unfiltered samples and for safety reasons (to prevent clogging), the samples were filtered for use on FlowCAM). Intergeneric classification was performed manually, and the distribution of classes is given in Suppl. Table 1. In total, the dataset included 972 images.

The same procedure was conducted to acquire intrageneric data of *Microcystis* spp. Images were recorded from 168 Mesocosm samples, out of which 69 samples were positive for the presence of *Microcystis* spp. The overall dataset included 119,135 images of *Microcystis* spp. Excluding *Sheaths* and *Non-colonial clusters* images, there were 70,305 images of colonial *Microcystis* separated into five classes based on the previous section's classification. The D1_17/09/2019 sample (collected from Mesocosm tank D1 in 17/09/2019) was used for training and test datasets as it contained a high proportion of all five *Microcystis* morphospecies in the amount of 5068 images; the detailed data distribution can be found in Suppl. Table 1. The image recordings from D2_09/03/2019 sample (collected from Mesocosm tank D2 on 09/03/2019) was used to assess intrageneric classifiers in a different dataset with 2,552 images of colonial *Microcystis.*

### Feature extraction and evaluation of filter sets

The imaging and cytometric data from LMWE samples were acquired using the FlowCAM instrument. To achieve an even distribution of representatives, 150 images were randomly moved from the Classification Window to Open view in the VisualSpreadsheet software. So, we used 150 representative images for each of the abundant phytoplankton species (*M. aeruginosa*, *M. ichthyoblabe*, *M. novacekii*, *M. wesenbergii*, *M. smithii*, *Cryptomonas*, and *Micractinium*) to train VisualSpreadsheet software to differentiate listed phytoplankton taxa. Then, 25 or 50 images were randomly selected as training data (further referred to as “25” and “50”), and the VisualSpreadsheet software generated an initial set of classification parameters specific for each species based on selected images. After auto-filtering, there were 48 image features left, which are based on five different categories: size, shape, texture, gray-scale signal, and color signal measurements.

The Filter Dialog box contained image features and their ranges between the minimum and maximum values. The filter sets were reduced by systematic selection to leave the minimal number of features until the filter set's accuracy started deviating significantly. In other words, the procedure was performed till the overall Accuracy value reached 0.75 and below, according to Eq. (1). The changes in filter sets resulted in an increase in the true positive rate with a drawback of decrease in the true negative rate. The changes in the true positive rate were recorded for each filter set, and they are compared in Suppl. Fig. 1. Shortlisted particle properties were saved in a filter format file. The value ranges were separately recorded for the selection of “25” and “50” images for training. These value ranges provide the basis for selection of particles/images in the dataset. It was decided to include the intersecting ranges between “25” and “50” to create the third type of filter set, named as “Intersection”. As this method is based on selected images, the produced classifier is equivalent to selecting more images ($50\le$ *X* $\le 75$). It was done to remove false positive results and increase the overall accuracy of the classification. So, the highest min. value and lowest max. value were taken manually to decrease the range for each parameter.

1 $$Accuracy = \frac{True\; Negative\; results + True\; Positive\; results}{{Total \;\;number \;of \;results}}$$

Equation (1): Equation for Accuracy that was used to leave the most important particle properties.

The above-mentioned filter sets were saved in filter format file and used for classification and further evaluation of the test dataset that excluded images from training dataset. The results of the test classification were recorded and used to construct a confusion matrix. Finally, the performance of the classification was evaluated based on reliability (precision) and accuracy of each classifier according to the procedure adapted from Aldenhoff et al.^45^.

Hellinger distance (HD) was used to identify any dataset shifts between the training dataset ($T$) and the test dataset ($X$). Equation (2) was adapted from the work of Cieslak and Chawla^46^. The minimum value for HD is 0, which is mainly observed when datasets are identical. The “1/$\sqrt{2}$” was added to change the maximum value of the HD from $\sqrt{2}$ (approximately 1.41) to 1.0.

2 $$Hellinger \,distance \left(X, T\right)= \frac{1}{s}\sum_{f=1}^{s}{HD}_{f}(X, T)=\frac{1}{s}\sum_{f=1}^{s}\frac{1}{\sqrt{2}}\sqrt{\sum_{k=1}^{c}{\left(\sqrt{\frac{{X}_{f,k}}{X}}-\sqrt{\frac{{T}_{f,k}}{T}}\right)}^{2}}$$

Equation (2): Equation for Hellinger distance between datasets X and T, where *s* represents the number of different filter sets used, ${HD}_{f}(X,T)$ is the Hellinger distance for a given filter set ($f$), $c$ is the number of classes (bins) used in classification method, $X$ is the total number of representative images in the dataset, and ${X}_{f,k}$ is the number of classification matches with the feature set $f$ that belongs to the class (or bin) $k$ (the same definitions are applied in the training dataset $T$).

Since we used test dataset with an uneven distribution of samples (Suppl. Table 1), the difference-based method was applied. The error for each class was identified using the equation for symmetric mean absolute percentage error (SMAPE), according to Eq. (3). This can be used as a clear indication of classification performance depending on the class^47^. The SMAPE analysis was performed to identify the error for each classification bin.

3 $$SMAPE ({c}_{i})= \frac{1}{s}\sum_{f=1}^{s}\frac{\left|{X}_{{f, c}_{i}}-{X{^{\prime}}}_{{f,c}_{i}}\right|}{{X}_{{f, c}_{i}}+{X{^{\prime}}}_{{f,c}_{i}}}$$

Equation (3): Equation for symmetric mean absolute percentage error (SMAPE) for each class, where ${X}_{{f, c}_{i}}$ is the actual value of the number of representatives for class ${c}_{i}$ using the filter set $k$, and ${X{^{\prime}}}_{{f,c}_{i}}$ represents the forecast value.

Bray–Curtis dissimilarity (Eq. 4) and Kullback–Leibler Divergence (Eq. 5) were used as metrics of the overall performance. In both approaches, the zero value indicates that the generated forecast distribution is fully identical with the actual data. The Bray–Curtis dissimilarity determines dissimilarity between the actual data and the data predicted by filter sets using their relative abundance data^47^. The Kullback–Leibler Divergence uses probability distributions to perform natural measures of relative entropy^46^. These values were calculated to assess the performance of the intergeneric and intrageneric (*Microcystis* morphospecies) approaches.

4 $$\mathrm{Bray }-\mathrm{Curtis\, dissimilarity}=\frac{\sum_{k=1}^{c}\left|{X}_{k}-{X{^{\prime}}}_{k}\right|}{\sum_{k=1}^{c}{X}_{k}+{X{^{\prime}}}_{k}}$$

Equation (4): Equation for Bray–Curtis dissimilarity for the overall performance metric, where ${X}_{k}$ is the actual value in the bin $k$, and ${X{^{\prime}}}_{k}$ represents the predicted value using the filter set $k$.

5 $$\mathrm{Kullback}-\mathrm{Leibler \,Divergence }\left(\mathrm{X}\parallel {\mathrm{X}}^{\mathrm{^{\prime}}}\right)= \sum_{k=1}^{c}\left[\frac{{X}_{k}}{\sum_{k=1}^{c}{X}_{k}}\times \left(\mathrm{log}\frac{{X}_{k}}{\sum_{k=1}^{c}{X}_{k}}-\mathrm{log}\frac{{X{^{\prime}}}_{k}}{\sum_{k=1}^{c}{X{^{\prime}}}_{k}}\right)\right]$$

Equation (5): Equation for Kullback–Leibler Divergence, where ${X}_{k}$ is the actual value in the bin $k$, and ${X{^{\prime}}}_{k}$ represents the predicted value using the filter set $k$.

## Results

### Classification parameters for each morphological class

Classification parameters were extracted using the feature finder tool in VisualSpreadsheet software. The description and possible range for each acquired particle property are summarized in Table 1. The selected most important particle properties are listed in Table 2. The upper table (Table 2A) summarizes particle properties with corresponding value ranges for intergeneric classification between three different genera, *Cryptomonas, Micractinium,* and *Microcystis*. The lower table (Table 2B) includes the same information for intrageneric classification of the five *Microcystis* morphospecies. For the “25” or “50” images selected as training data, the ranges are shown as a minimum and maximum values. The “intersection” filter with narrowed value ranges is included to assess intercepting images between “25” or “50”.

**Table 1:** List of particle properties with corresponding type, description and possible value range. (modified from FlowCAM user manual).

Particle properties	Type	Descriptions	Value range for X
Average Blue	Color	Average pixel value for blue color plane	X ∈ [0, 255];
Diameter (ABD)	Size	Circle-based diameter that equal to ABD Area	X > 0;
Edge gradient	Texture	Average pixels intensity of outside border of a particle after an application of Sobel Edge Detect convolution filter	X ∈ [0, 255];
Intensity	Grayscale	Average grayscale value of pixels of a particle (grayscale sum / number of particle pixels)	X ∈ [0, 255];
Length	Size	Maximum value of 36 feret measurements	X > 0;
Perimeter	Size	Total length of edges including edges of any hole	X > 0;
Ratio red/blue	Color	Ratio between Average Red and Average Blue	X ≥ 0;
Ratio red/green	Color	Ratio between Average Red and Average Green	X ≥ 0;
Roughness	Shape	Unevenness/irregularity of a particle's surface, defined as the ratio between perimeter and convex perimeter. Larger values have a non-convex perimeter and/or interior holes	X ≥ 1; X = 1 for a filled shape with convex perimeter;
Sigma intensity	Grayscale	Standard deviation of particle’s grayscale values	X ≥ 0

**Table 2:** Set of particle properties within each filter set for intergeneric classification (A) and intrageneric classification (B).

A						
Particle property	25 selected		50 selected		Intersection	
	Min	Max	Min	Max	Min	Max
*Cryptomonas*						
Diameter (ABD) (μm)	8.23	11.53	8.22	12.72	8.23	11.53
*Micractinium*						
Diameter (ABD) (μm)	16.19	40.52	15.85	40.52	16.19	40.52
Intensity	79.20	105.85	73.64	105.85	79.20	105.85
*Microcystis novacekii*						
Diameter (ABD) (μm)	45.35	85.69	33.48	83.89	45.35	83.89
Intensity	49.12	92.47	40.55	97.59	49.12	92.47

B						
Particle property	25 selected		50 selected		Intersection	
	Min	Max	Min	Max	Min	Max
*M. aeruginosa*						
Average blue	76.28	101.01	76.08	97.85	76.28	97.85
Edge gradient	99.93	159.87	96.11	159.87	99.93	159.87
Perimeter (μm)	391.00	1620.14	391.00	1460.73	391.00	1460.73
Ratio red/blue	1.15	1.28	1.15	1.30	1.15	1.28
Sigma intensity	23.72	34.09	23.68	35.70	23.72	34.09
*M. ichthyoblabe*						
Average blue	72.02	96.30	77.64	96.30	77.64	96.30
Intensity	78.76	110.43	83.81	110.43	83.81	110.43
Length (μm)	56.85	138.37	60.17	128.39	60.17	128.39
Ratio red/blue	1.17	1.32	1.15	1.32	1.17	1.32
Roughness	1.42	4.16	1.38	4.16	1.42	4.16
Sigma intensity	22.14	28.37	21.58	30.97	22.14	28.37
*M. novacekii*						
Intensity	48.36	80.51	46.45	74.81	48.36	74.81
*M. smithii*						
Intensity	94.55	117.32	93.98	120.00	94.55	117.32
Ratio red/green	1.17	1.35	1.16	1.39	1.17	1.35
Roughness	2.19	10.70	1.58	10.70	2.19	10.70
*M. wesenbergii*						
Average blue	65.24	85.13	61.31	85.13	65.24	85.13
Intensity	73.59	98.04	67.25	98.04	73.59	98.04
Ratio red/blue	1.19	1.36	1.19	1.39	1.19	1.36
Ratio red/green	1.17	1.23	1.15	1.27	1.17	1.23
Sigma intensity	26.89	43.70	26.10	47.12	26.89	43.70

### Evaluation of filter sets in high-throughput data set

Generated sets of particle properties were examined on the test dataset that exhibited an uneven distribution (more detailed in Suppl. Table 1). The image collection covered only described genera-level and morphospecies-level classification. In other words, images of other species were excluded from the comparison of selected types of data.

The “Intersection” filter setting with narrowed ranges was used as the representative data. Results of semi-automated classification, “predicted” by classifiers, were compared with the manual classification data, that is considered as “True label”. The confusion matrices were constructed based on true positive results and misclassifications by calculating precision and false discovery rate, respectively (Table 3A,B). Overall performance for intergeneric and intrageneric classification in percentage is provided in Tables 3C,D, and more detailed information on the other two filter settings, when “25” and “50” images were used for training, can be found in Suppl. Tables 2 and 3.

**Table 3:**
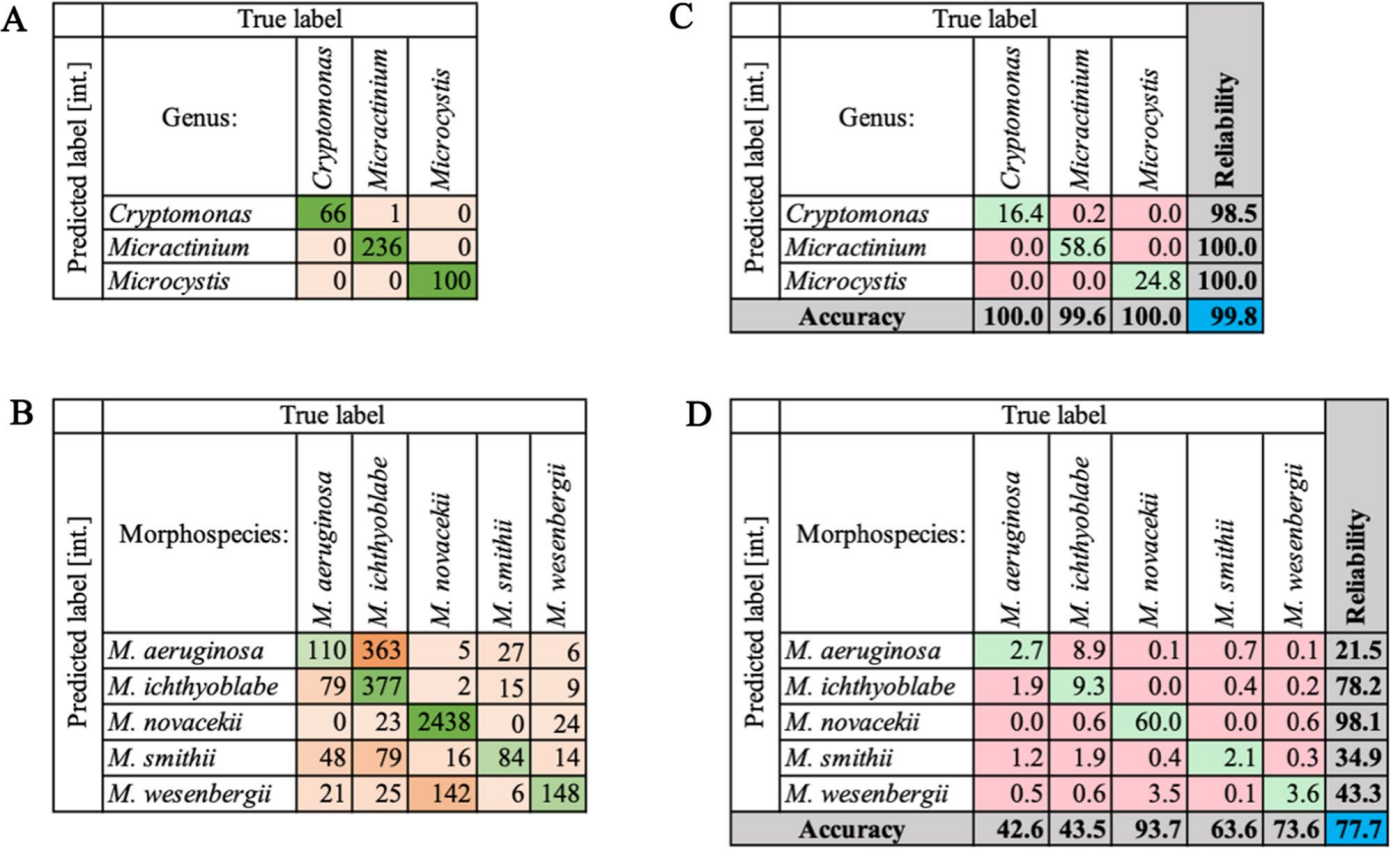
The results of classification using intersecting ranges for the intergeneric classification of three classes (upper row) and the intrageneric classification of five *Microcystis* morphospecies (lower row).

(A, B) Numbers represent results when corresponding classifier was applied. Confusion matrices for used filter sets with precision values for correct predictions (highlighted in green) and false discovery rate for misclassifications (highlighted in orange). The coloring was assigned depending on relative frequency in each column. (C, D) Percentage values for accuracy and reliability (precision) of used methods. Overall performance for intergeneric and intrageneric classification in percentage is highlighted in blue.

The classification of intergeneric *Cryptomonas*, *Micractinium*, and *Microcystis *images was performed using only two-particle properties. Setting ranges for diameter (ABD) and intensity was enough to discriminate between one and the two other classes with an overall 96–100% performance for these classifiers. *Cryptomonas* has the smallest cell size (8–13 μm) of the three genera, and size was consequently used as a feature. On selected 25 images, the diameter ranges of the *Micractinium* and *Microcystis *filter sets did not intersect. However, when the training set was increased to 50, the intensity feature's inclusion became necessary to ensure accurate classification, implying combined use of the size and signal strength feature categories for the larger filter sets. The confusion matrices showed a low misclassification rate with an overall 96–100% performance for the dataset of 971 images. The differences between the three species (genera) were sufficient for the VisualSpreadsheet software to perform useful classification.

The performance of the intrageneric *Microcystis *spp. classification was considerably lower than for the intergeneric classification. Firstly, the filtering pipeline for classification included a wider variety of particle properties. Since the colony size of *Microcystis* spp. colonies varied, the basic diameter (ABD) parameter was not applied. However, the size-based parameters of perimeter and length were used for differentiation of *M. aeruginosa* and *M. ichthyoblabe*, respectively. In the training dataset, the false discovery rate was as low as 0.47. However, it was increased to 0.71 when *M. aeruginosa* classifier was applied to the test dataset. Additionally, Shape features were used in classifiers for *M. aeruginosa*, *M. ichthyoblabe*, and *M. smithii*. The initial Shape classifier had Edge gradient parameter due to a semi-transparent halo's appearance around *M. aeruginosa* colonies. The second shape feature was Roughness, which has increased values when bigger interior holes are present. In our prediction system, the classifier for *M. smithii* had a higher value range between 1.58 and 10.70 compared to *Microcystis ichthyoblabe* (1.38–4.16). The Roughness particle property differentiated the other two colonial morphospecies.

All other particle properties were based on Signal strength, namely Average Blue, Intensity, Ratio Red/Blue, Ratio Red/Green, and Sigma Intensity. The classifier for *M. wesenbergii* had a combination of Signal strength features, including Intensity, Average Blue (average value for blue color pixels), and Sigma Intensity (standard deviation of particle’s grayscale values). These features could not adequately filter out *M. novacekii* images, resulting in a lower accuracy and reliability values of 73.6% and 43.3%, respectively. However, these classifier features were efficient in elimination of *M. aeruginosa*, *M. ichthyoblabe*, and *M. smithii* images from *M. wesenbergii* classifier bin, which resulted in considerably low false discovery rate of 0.02–0.07. Finally, *M. novacekii *was identified through the single gate of Intensity feature because the small value range did not intersect with other morphospecies, and provide a relatively high precision of 98.1%.

Since the test dataset was imbalanced with an uneven distribution of representatives, balanced accuracy was calculated for each filter set application by averaging the true positive rate and true negative rate. Different validation methods were used to examine both the training dataset and the test dataset for each filter set and the summary for the calculations is given in Table 4.

**Table 4:** Summary of the evaluation of filter sets using balanced accuracy, Hellinger distance and SMAPE, Bray–Curtis dissimilarity, and Kullback–Leibler Divergence.

Filter set used for classification	Hellinger distance x ∈ [0,1]	Balanced accuracy (%)	SMAPE (%)	Bray–Curtis dissimilarity x ∈ [0,1]	Kullback–Leibler Divergence x ≥ 0
**Intergeneric classification**					
*Cryptomonas*	0.13	90.13	3.60	0.128	0.0006
*Micractinium*	0.10	87.26	5.04		
*M. novacekii*	0.07	91.32	3.17		
**Intrageneric classification**					
*M. aeruginosa*	0.19	73.2	17.24	0.145	0.0281
*M. ichthyoblabe*	0.15	71.3	16.44		
*M. novacekii*	0.24	90.7	2.75		
*M. smithii*	0.17	77.7	11.05		
*M. wesenbergii*	0.16	74.3	9.68		

The results show that intergeneric classifications between three different genera have lower Hellinger distance, which indicates that small data shifts can influence the performance of filter sets. The data shift value for morphospecies (intrageneric) classification was considerably higher than for the intergeneric classification, which affected their classification accuracy. However, there was no strong linear correlation between Hellinger distance and balanced accuracy, especially in the intergeneric classification.

For both the intergeneric and intrageneric approaches, the accuracy values for *M. novacekii* were around 91%. Other genera (*Cryptomonas* and *Micractinium*) had balanced accuracy values of 87–90%, while the other four *Microcystis* morphospecies has lower values of 73–78%. The SMAPE analysis exhibited an error percentage of ≤ 5.0% for the intergeneric classification, rising to 17.2% for the *M. aeruginosa* filter set in the morphospecies classification.

The results of the two classification approaches were evaluated by Bray–Curtis dissimilarity, and the intrageneric classification had a higher dissimilarity (0.145) than the classification between genera (0.128). The results were checked by calculating Kullback–Leibler Divergence (KLD) to compare the probability distributions of predicted data and actual data. The calculations also indicated a lower KLD value of 0.0006 for the intergeneric classification compared to the intrageneric classification value of 0.0281. In other words, the classification information lost using the method of genera classification is lower than when using the classification between morphospecies.

### Dynamics of a seasonal *Microcystis* bloom succession in LMWE-2019 mesocosm

**Figure 3:**
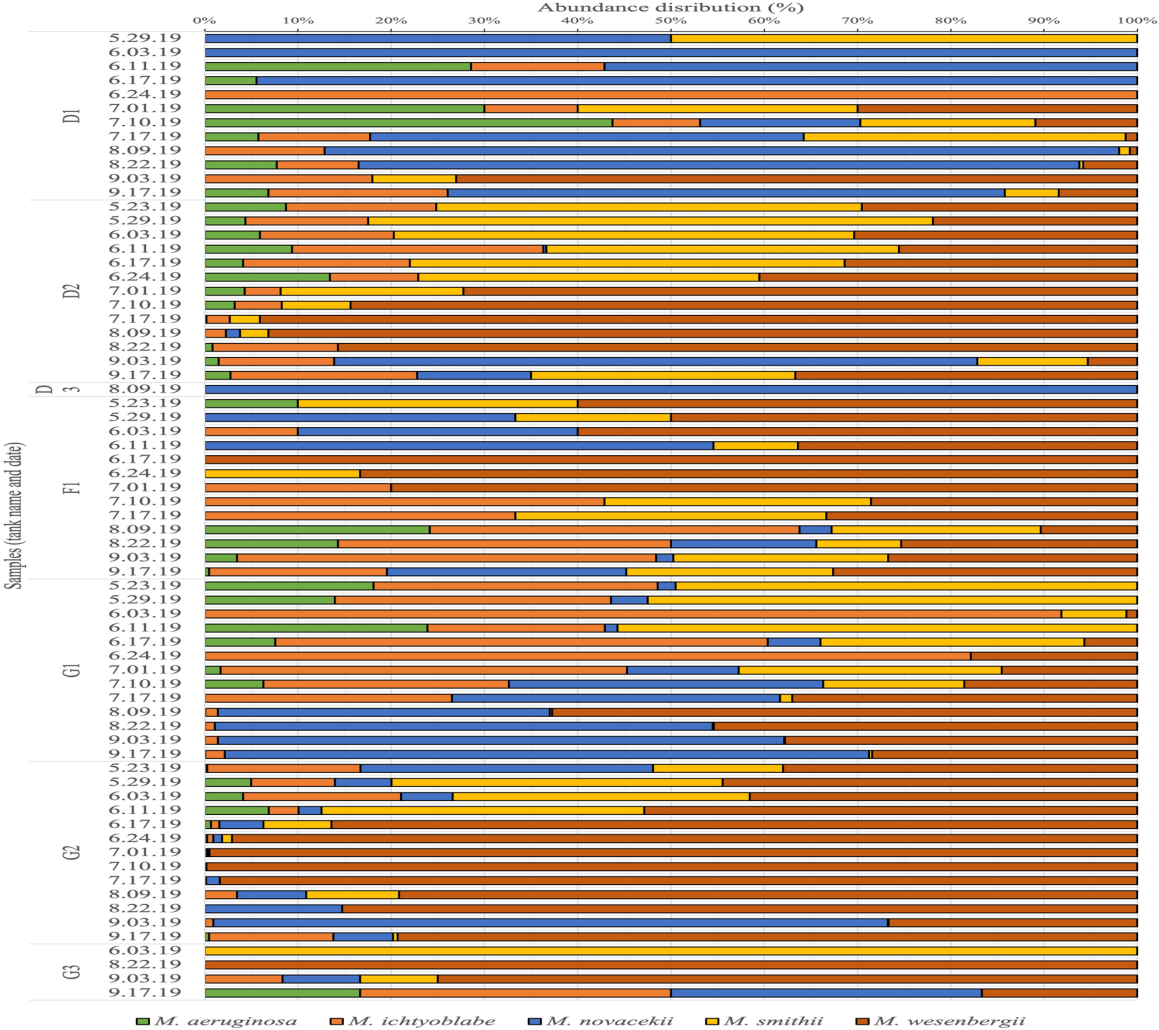
The distribution of *Microcystis* morphospecies in samples from the LMWE experiment in 2019. Grouping is based on type of tank and the date when the samples were taken.

**Figure 4:**
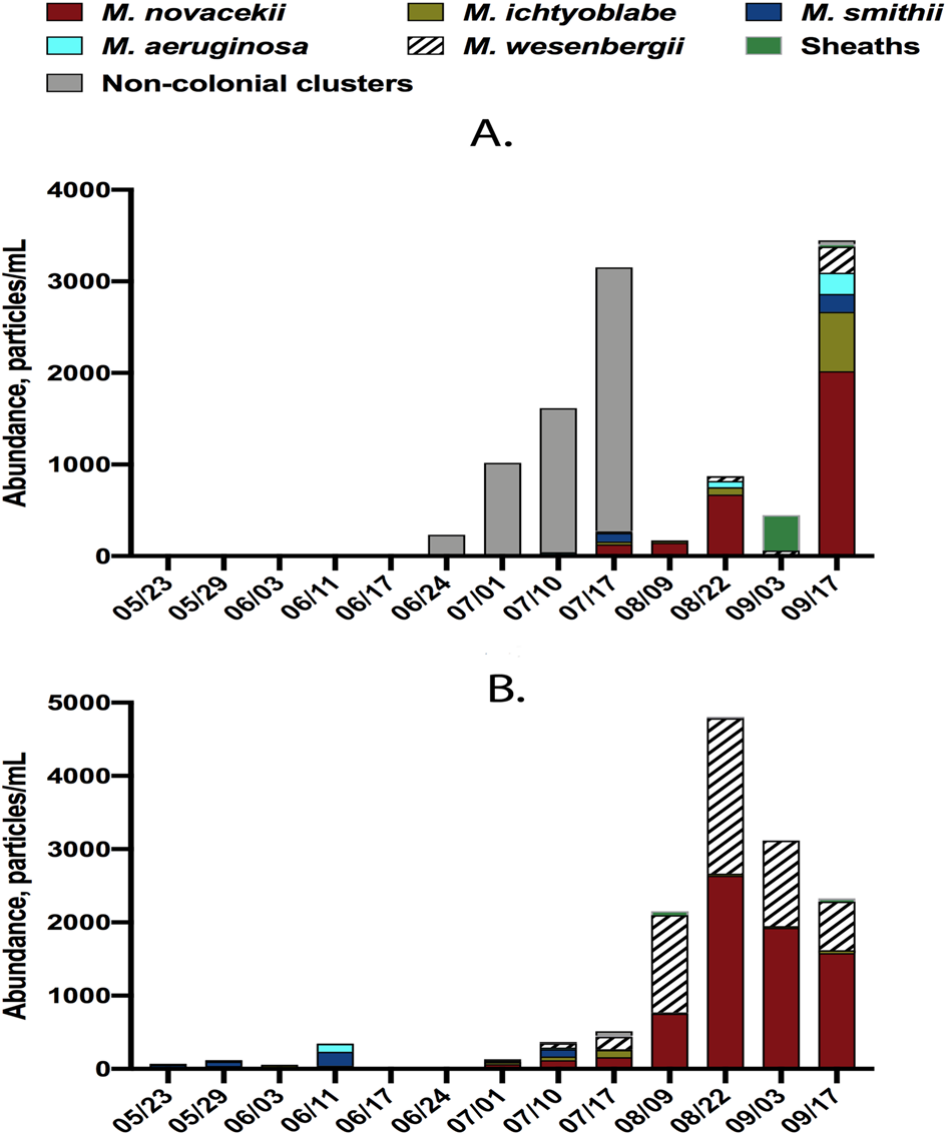
Seasonal changes of abundances of *Microcystis* spp. colonial morphoforms and water temperature from May to September 2019 in mesocosm tanks D1 (**A**) and G1 (**B**).

The results of dominant species analysis by light microscopy in the tanks containing *Microcystis* spp. are listed in Suppl. Table 4; and often included *Micractinium* spp. (detailed in Suppl. Table 4) or *Cryptomonas* spp. (D3, F3, G3 tanks, at different dates). The semi-automatic classification based on well-balanced training sets from *Microcystis* seasonal bloom provided a high level of intergeneric accuracy (96–100%) (in comparison with *Micractinium* spp. and *Cryptomonas* spp.) but relatively low intrageneric accuracy (67–78%). The percentage distribution of colonial *Microcystis* morphospecies in samples from the LMWE experiment in 2019 is presented in Fig. 3 below. Importantly, there was a sequential appearance of the *Microcystis* spp. morphotypes, and also some *Microcystis* morphotypes (*M.aeruginosa, M.novacekii, M.smithii*) completely disappeared during certain periods of time and re-appeared later Fig. 4A and B.

## Discussion

This study demonstrated the proof-of-concept of using a machine learning approach in the analysis of colonial morphospecies of *Microcystis*. The results described here are based on previous researchers' work demonstrating the application of machine learning in the identification and counting of different taxa of plankton organisms^1,2,5,7,15^. The current gold standard for phytoplankton taxonomy is light microscopy of algal samples; however, there is a huge interest to apply semi-automated and automated approaches for *Microcystis* colonial forms classification. Light microscopy's biggest disadvantages are the extensive training and time period required for a taxonomist to become a proficient expert, the high cost of training, and the large component of manual work involved. Although sequencing and the following molecular biological identification have become more popular in recent years, microscopy and visual morphological analysis remain the most important and widely available tools. In the context of saving time during taxonomic analysis, imaging cytometers constitute a faster and efficient way to receive the morphological information required for taxonomic identification^5^. The imaging cytometer in our study was a FlowCAM instrument used by many research groups worldwide^5,8,12,19^.

In recent years, automatic classification of plankton has attracted increasing attention, with the development of methods including both handcrafted features^48–50^ and deep learning architectures^51–54^. The former was used for semi-automatic classification by Gorsky et al.^3^, who applied the ZooProcess and Plankton Identifier software for feature extraction and zooplankton taxonomic characterization. The latter, being based on convolutional neural networks, used input images to extract features for several classifiers, but this was a task that required a considerably higher number of annotated images as training datasets for each class^55^. However, the authors found it difficult to create a well-balanced training dataset for deep learning from natural samples with both high diversity and a high abundance of plankton taxa. The images extracted from field samples often showed a natural class imbalance of phytoplankton taxa. For example, Lee et al.^51^ used the WHOI-Plankton database with 3 million plankton images, where > 90% of all images were annotated for only 5 different classes. In the recent study by Kerr and co-authors^12^, the class imbalance issue was addressed by constructing deep learning algorithms in a collaborative model to achieve the classification of under represented classes found in FlowCAM images. However, this prediction model showed poor performance in certain minority classes. If the non-target training instances heavily outnumber the target classes' training instances, the deep learning algorithms can be ineffective in determining class boundaries. Several studies demonstrate that balanced image distributions yield the best performances^56–58^. We had the advantage of observing seasonal blooms in the mesocosm samples, which helped create well-balanced training sets of *Microcystis* morphospecies for use in a semi-automated classification approach. In the 2019 LMWE experiment, we followed a *Microcystis* seasonal bloom represented by a changing ratio of colonial morphospecies at different dates (Fig. 3). This allowed us to create class-balanced training sets by choosing time points with sufficient amounts of all five *Microcystis* morphospecies. It is a first attempt to apply a semi-automatic algorithm for intrageneric analysis of colonial *Microcystis*, the majority of previous studies being focused on the analysis of colonial phytoplankton taxa at genus level^10,12,53^ or used for analysis training sets build with single-celled *Microcystis* laboratory cultures^9,11^.

Here, we presented an identification logic and statistical evaluation of the accuracy and reliability of the approach used for the classification of five colonial morphospecies of *Microcystis* available from a seasonal mesocosm experiment. To verify a machine learning approach for intergeneric classification, we also used plankton from different genera, namely, unicellular *Cryptomonas* and colonial *Micractinium,* available from the mesocosm experiment plankton samples taken during the 2019 season*. Cryptomonas* was represented by brown-green colored asymmetric cells with a transparent membrane on the outside and an average size of about 40 μm. It is non-toxic freshwater algae with two flagella and is usually consumed by zooplankton^44^. The representative from the second algal genus was the colonial green algae *Micractinium*, which has proteinaceous spines to prevent grazing by planktonic rotifers^59^. Based on microscopy analysis, both algae were dominant or co-dominant with *Microcystis* in LMWE-2019 tanks at many dates (Suppl. Table 4 for *Micractinium* and F3 tanks for *Cryptomonas* spp.). We developed a machine learning approach based on the simple brightfield-related morphological descriptors that demonstrated high performance at the intergeneric level of phytoplankton taxa with a training set of image samples derived from different time points of the 2019 LMWE season. Overall, the accuracy of intergeneric classification of *Microcystis* spp. in the mesocosm samples compared to other colonial and/or unicellular algae showed high performance of 96–100%, stressing the value of using minimized filter sets including 1–2 features. However, this semi-automated classification demonstrated 65–75% accuracy for intrageneric morphospecies within colonial *Microcystis* spp. This type of classification required significantly more filter descriptors (up to 5 particle properties)*.* Nevertheless, the obtained results are comparable with those of analysis by human taxonomists, which, according to Culverhouse and co-authors^14^, is between 67 and 83%. It means that it is possible to evaluate the automatically significant percentage of acquired during seasonal bloom *Microcystis* images and save 70–80% of researcher time.

By contrast, the suggested machine learning approach using well-balanced training sets covering the whole seasonal bloom demonstrated a higher level of accuracy of up to 93% for intrageneric differentiation of *Microcystis* morphospecies, if a training set was created and applied to the images of the five algal forms taken as they occurred at a one-time point in the samples during the bloom. However, a set of classification parameters tends to be less optimal to a particular tank and sampling date. It has less accuracy when applied to other sample sets (detailed description is provided in Suppl. Table 5). We hypothesize that the decrease in the accuracy can be explained by a significant level of colonial phenotypic variability, i.e., high heterogeneity of toxic and non-toxic *Microcystis* morphospecies^60–62^ during the seasonal bloom. *Microcysti*s heterogeneity shows up, evidenced by differences in image features patterns encountered when data sampling dates are separated by a few weeks.

The described machine learning approach was applied to produce a long-term dataset aimed to understand the colonial *Microcystis* development in relation to environmental factors (manuscript in preparation). The obtained data revealed a sequential seasonal disappearance/reappearance of the certain colonial *Microcystis* morphoforms (Figs. 3 and 4A,B). Morphological variability of *Microcystis* colonies induced by laboratory conditions have been described recently by different groups^63,64^. Similar observations of sequential changes and disappearance of certain colonial *Microcystis* morphologies were reported in Lake Taihu study^65^. Together these observations and our results obtained with machine learning analysis of colonial *Microcystis* are supporting the hypothesis of main transition pathways of colonial *Microcystis* morphoforms^61^. The classification of cyanobacteria strains that was done exclusively by morphological characteristics is not always sufficient^61,66,67^, and our observations emphasize the early formulated suggestions that previously distinct morphospecies may belong to single species^68^.

Colony formation of *Microcystis* is thought to contribute to the global success of this genus in freshwater ecosystems^69,70^. With an increase of environmental problems related to climate change and water scarcity^71^, we need to understand better the factors and mechanisms affecting *Microcystis* colonial forms evolution and dominance. This study provides a useful approach for quantitative analysis of *Microcystis* diversity.

## Conclusions

The estimation of speed/type for phenotypic changes in colonial *Microcystis* requires a high spatial and temporal resolution, and mesocosm studies of seasonal *Microcystis* spp. succession together with semi-automated machine learning algorithm of colonial forms analysis may provide much more detailed and less prone to user bias analysis. Morphological analysis of phytoplankton along time and at recording seasonal changes of single species represents an important tool to study dynamics of aquatic ecosystems^72,73^. Our results suggest that by combining intrageneric classification with the relatively simple set of descriptors in imaging flow cytometry, we can provide an opportunity to examine the colonial morphoforms of *Microcystis* at a higher resolution and temporal level during seasonal bloom. Although previous studies have developed machine learning and deep learning approach to classify plankton^1–12^, our study is the first to differentiate colonial morphoforms of freshwater *Microcystis* at the intrageneric level. The accuracy of the approach is raising to experienced human taxonomists' performance level, thereby reducing the time of analysis and subjectivity. As one of the significant outcomes of this work, such results further highlights a high level of *Microcystis* spp. heterogeneity during a seasonal bloom and support the hypothesis of main transition pathways of colonial *Microcystis* morphoforms. The classification algorithm’s accuracy depends on the increased diversity of images features, which can be enriched in the future by including a variety of fluorescence-correlated morphological parameters in the filter sets. We expect that automated methods will be increasingly used in the future, allowing early detection of toxic morphospecies of colonial *Microcystis* and other harmful algae.

## Supplementary Information

Supplementary Information.
