# Supplementary material for: Semi-automated classification of colonial Microcystis by FlowCAM imaging flow cytometry in mesocosm experiment reveals high heterogeneity during seasonal bloom
Source: Sci Rep. 2021 Apr 30;11:9377. doi: 10.1038/s41598-021-88661-2 (PMC8087837; doi:10.1038/s41598-021-88661-2)
Supplement: Supplementary file 1 — Supplementary Information. [file 41598_2021_88661_MOESM1_ESM.docx]

**Semi-Automated Classification of Colonial *Microcystis* by FlowCAM Imaging Flow Cytometry in Mesocosm Experiment Reveals High Heterogeneity during Seasonal Bloom**

Yersultan Mirasbekov^1,#^, Adina Zhumakhanova^1,#^, Almira Zhantuyakova^1,#,§^, Kuanysh Sarkytbayev^1,2^, Dmitry V. Malashenkov^3^, Assel Baishulakova^1^, Veronika Dashkova^1,4^, Thomas A. Davidson^5^, Ivan A. Vorobjev^1,2^, Erik Jeppesen^5,6,7,8^, Natasha S. Barteneva^1,9,*^

^#^These authors contributed equally: Yersultan Mirasbekov, Adina Zhumakhanova and Almira Zhantuyakova.

^*^Correspondence to: natalie.barteneva@nu.edu.kz

Supplementary Files: 1 figure and 5 tables

**Legends to Supplementary Figures:**

**Supplementary Figure 1.** Comparison of the true positive rates before and after systematic selection of particle properties.

**Legends to Supplementary Tables:**

**Supplementary Table 1.** Number of representative images for each class after intergeneric classification (left) and intrageneric classification (right).

**Supplementary Table 2.** The results of classification using classifier of “25” selected images for the intergeneric classification of three classes (upper row) and the intrageneric classification of five morphospecies (lower row). (A-B) Confusion matrices for used filter sets with precision values for correct predictions (highlighted in green) and false discovery rate for misclassifications (highlighted in orange). (C-D) Percentage values for accuracy and reliability (precision) of used methods. Overall performance for intergeneric and intrageneric classification in percentage is highlighted in blue.

**Supplementary Table 3.** The results of classification using classifier of “50” selected images for the intergeneric classification of three classes (upper row) and the intrageneric classification of five morphospecies (lower row).

**Supplementary Table 4.** Dominant phytoplankton species in LMWE-2019 experimental tanks.

**Supplementary Table 5.** The results of intrageneric classification using intersecting ranges on another sample. The sample was collected from Mesocosm tank D2 in 09/03/2019.

**Supplementary Figure 1.** Comparison of the true positive rates before and after systematic selection of particle properties.

**Supplementary Table 1**. Number of representative images for each class after intergeneric classification (left) and intrageneric classification (right).

| Name of genus | Number of representatives |  | Training set | Test dataset |
| --- | --- | --- | --- | --- |
| *Cryptomonas* | 232 |  | 150 | 82 |
| *Micractinium* | 469 |  | 150 | 318 |
| *M. novacekii* | 271 |  | 150 | 121 |
| Total | 972 |  | 450 | 521 |
|  |  |  |  |  |
| Name of *Microcystis* spp*.* morphotypes | Number of representatives |  | Training set | Test dataset |
| *M. aeruginosa* | 346 |  | 150 | 196 |
| *M. ichthyoblabe* | 975 |  | 150 | 825 |
| *M. novacekii* | 3028 |  | 150 | 2878 |
| *M. smithii* | 292 |  | 150 | 142 |
| *M. wesenbergii* | 427 |  | 150 | 277 |
| Total | 5068 |  | 750 | 4318 |

**Supplementary Table 2.** The results of classification using classifier of “25” selected images for the intergeneric classification of three classes (upper row) and the intrageneric classification of five morphospecies (lower row). (A-B) Confusion matrices for used filter sets with precision values for correct predictions (highlighted in green) and false discovery rate for misclassifications (highlighted in orange). (C-D) Percentage values for accuracy and reliability (precision) of used methods. Overall performance for intergeneric and intrageneric classification in percentage is highlighted in blue.


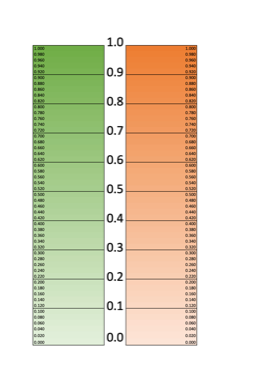

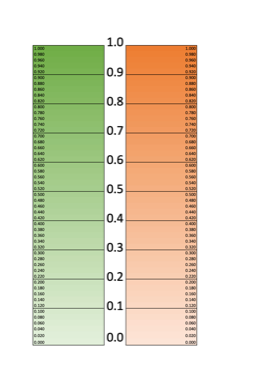
**
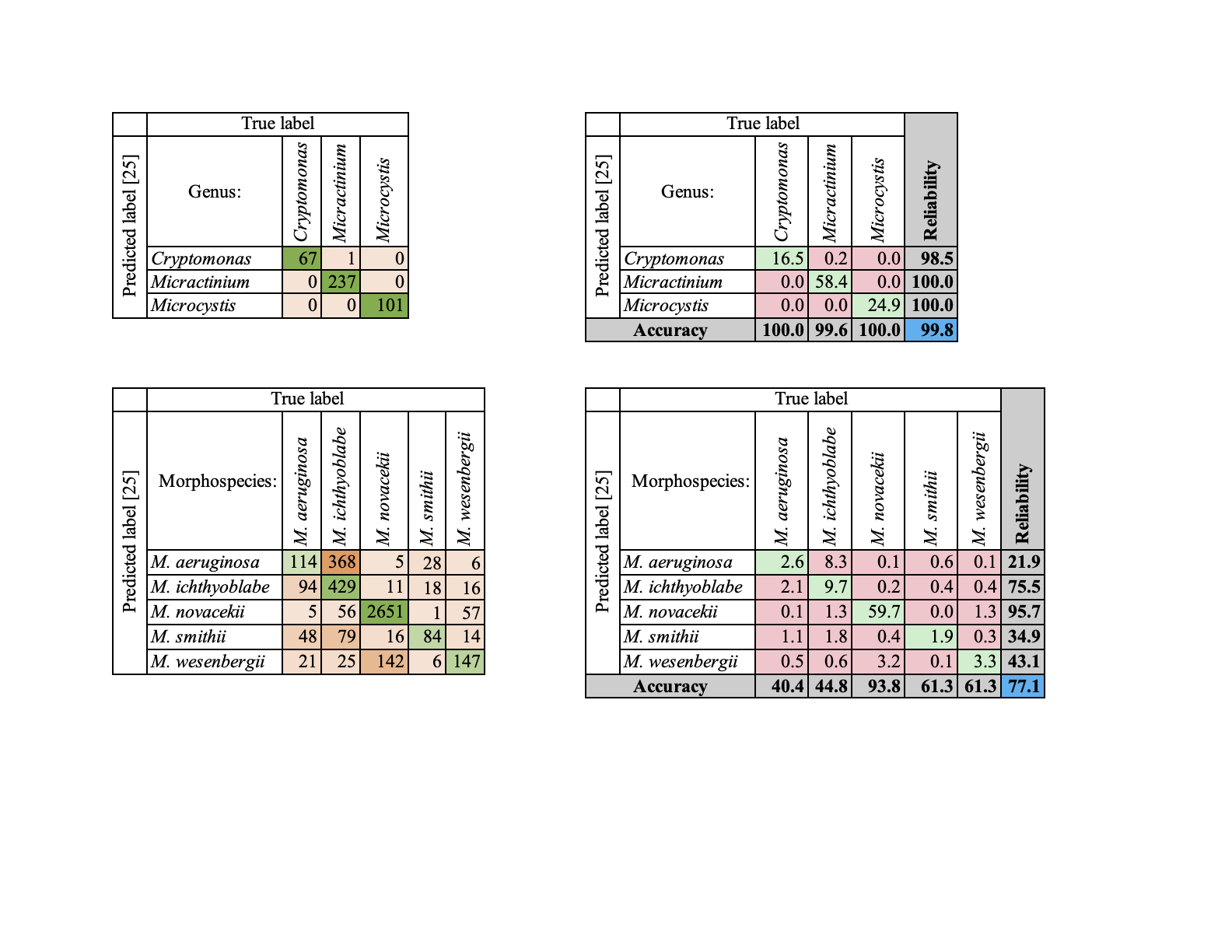
**

**C**

**D**

**A**

**B**

**Supplementary Table 3.** The results of classification using classifier of “50” selected images for the intergeneric classification of three classes (upper row) and the intrageneric classification of five morphospecies (lower row). (A-B) Confusion matrices for used filter sets with precision values for correct predictions (highlighted in green) and false discovery rate for misclassifications (highlighted in orange). (C-D) Percentage values for accuracy and reliability (precision) of used methods. Overall performance for intergeneric and intrageneric classification in percentage is highlighted in blue.


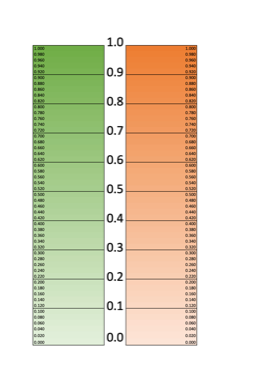

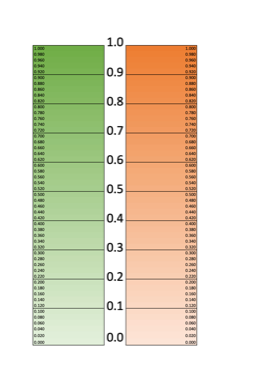

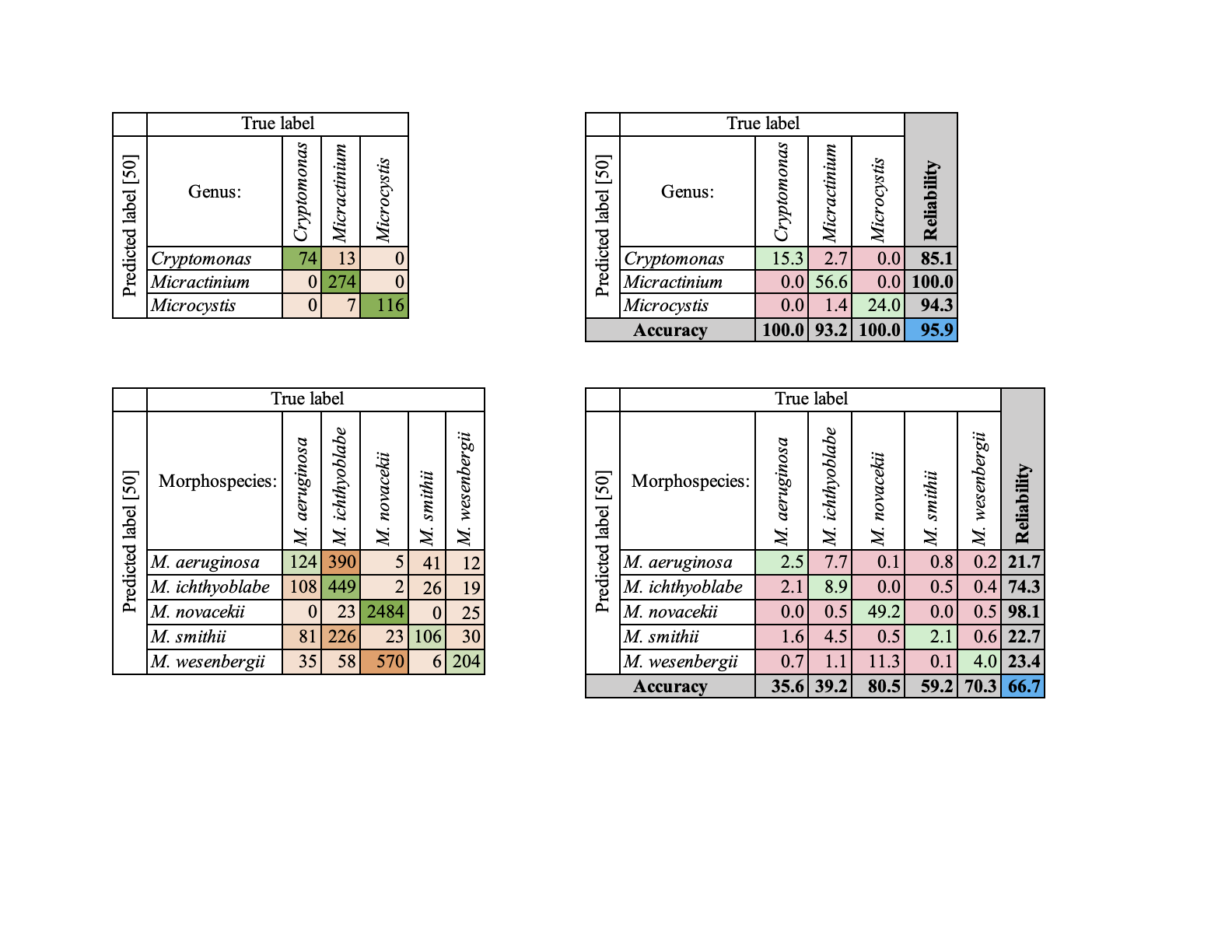


**A**

**B**

**C**

**D**

**Supplementary Table 4.** Dominant phytoplankton species in LMWE-2019 experimental tanks.

| Tank | Date | Dominant species | Subdominant species |
| --- | --- | --- | --- |
| D1 | 5/29/19 | *Pediastrum duplex* Meyen | *Microcystis* spp. |
|  | 6/03/19 | *Pediastrum duplex* Meyen | *Pseudanabaena* cf. *limnetica* (Lemmermann) Komárek |
|  | 6/11/19 | *Pediastrum duplex* Meyen |  |
|  | 6/17/19 | *Micractinium pusillum* Fresenius |  |
|  | 6/24/19 | *Micractinium pusillum* Fresenius |  |
|  | 7/01/19 | *Micractinium pusillum* Fresenius |  |
|  | 7/10/19 | *Micractinium pusillum* Fresenius |  |
|  | 7/17/19 | *Micractinium pusillum* Fresenius |  |
|  | 8/09/19 | *Micractinium pusillum* Fresenius | *Microcystis* spp. |
|  | 8/22/19 | *Micractinium pusillum* Fresenius |  |
|  | 9/03/19 | *Micractinium pusillum* Fresenius |  |
|  | 9/17/19 | *Micractinium pusillum* Fresenius |  |
| D2 | 5/23/19 | *Pediastrum duplex* Meyen | *Micractinium pusillum* Fresenius, *Pseudanabaena* cf. *limnetica* (Lemmermann) Komárek |
|  | 5/29/19 | *Pediastrum duplex* Meyen |  |
|  | 6/03/19 | *Pediastrum duplex* Meyen | *Microcystis* spp. |
|  | 6/11/19 | *Microcystis* spp. | *Pseudanabaena* cf. *limnetica* (Lemmermann) Komárek, *Stephanodiscus hantzschii* Grunow |
|  | 6/17/19 | *Microcystis* spp. | *Pseudanabaena* cf. *limnetica* (Lemmermann) Komárek, *Stephanodiscus hantzschii* Grunow |
|  | 6/24/19 | *Microcystis* spp. |  |
|  | 7/01/19 | *Microcystis* spp. |  |
|  | 7/10/19 | *Microcystis* spp. | *Pseudanabaena* cf. *limnetica* (Lemmermann) Komárek |
|  | 7/17/19 | *Microcystis* spp. |  |
|  | 8/09/19 | *Microcystis* spp. | *Pediastrum duplex* Meyen |
|  | 8/22/19 | *Micractinium pusillum* Fresenius |  |
|  | 9/03/19 | *Micractinium pusillum* Fresenius |  |
|  | 9/17/19 | *Pseudanabaena* cf. *limnetica* (Lemmermann) Komárek |  |
| D3 | 8/09/19 | *Micractinium pusillum* Fresenius |  |
| F1 | 5/23/19 | *Pseudanabaena* cf. *limnetica* (Lemmermann) Komárek | *Micractinium pusillum* Fresenius |
|  | 5/29/19 | *Micractinium pusillum* Fresenius | *Pediastrum duplex* Meyen, *Stephanodiscus hantzschii* Grunow |
|  | 6/03/19 | *Pseudanabaena* cf. *limnetica* (Lemmermann) Komárek, *Micractinium pusillum* Fresenius | *Pediastrum duplex* Meyen, *Stephanodiscus hantzschii* Grunow |
|  | 6/11/19 | *Micractinium pusillum* Fresenius, *Raphidocelis* cf. *sigmoidea* Hindák | *Pediastrum duplex* Meyen, *Stephanodiscus hantzschii* Grunow |
|  | 6/17/19 | *Pseudanabaena* cf. *limnetica* (Lemmermann) Komárek | *Pediastrum duplex* Meyen |
|  | 6/24/19 | *Pediastrum duplex* Meyen |  |
|  | 7/01/19 | *Pediastrum duplex* Meyen | *Desmodesmus armatus* (R.Chodat) E.Hegewald |
|  | 7/10/19 | *Pediastrum duplex* Meyen | *Desmodesmus armatus* (R.Chodat) E.Hegewald |
|  | 7/17/19 | *Pediastrum duplex* Meyen |  |
|  | 8/09/19 | *Desmodesmus armatus* (R.Chodat) E.Hegewald | *Choricystis* sp. |
|  | 8/22/19 | *Pediastrum duplex* Meyen | *Microcystis* spp., *Pseudanabaena* cf. *limnetica* (Lemmermann) Komárek |
|  | 9/03/19 | *Microcystis* spp. | *Micractinium pusillum* Fresenius, *Raphidocelis* cf. *sigmoidea* Hindák |
|  | 9/17/19 | *Microcystis* spp. |  |
| G1 | 5/23/19 | *Pediastrum duplex* Meyen |  |
|  | 5/29/19 | *Desmodesmus armatus* (R.Chodat) E.Hegewald |  |
|  | 6/03/19 | *Pediastrum duplex* Meyen | *Microcystis* spp., *Desmodesmus armatus* (R.Chodat) E.Hegewald |
|  | 6/11/19 | *Microcystis* spp. |  |
|  | 6/17/19 | *Pediastrum duplex* Meyen | *Microcystis* spp. |
|  | 6/24/19 | *Pediastrum duplex* Meyen | *Microcystis* spp. |
|  | 7/01/19 | *Microcystis* spp., *Pediastrum duplex* Meyen | *Pseudanabaena* cf. *limnetica* (Lemmermann) Komárek |
|  | 7/10/19 | *Microcystis* spp. | *Pediastrum duplex* Meyen, *Pseudanabaena* cf. *limnetica* (Lemmermann) Komárek |
|  | 7/17/19 | *Microcystis* spp. | *Pseudanabaena* cf. *limnetica* (Lemmermann) Komárek |
|  | 8/09/19 | *Microcystis* spp. |  |
|  | 8/22/19 | *Microcystis* spp. |  |
|  | 9/03/19 | *Microcystis* spp. |  |
|  | 9/17/19 | *Microcystis* spp. |  |
| G2 | 5/23/19 | *Micractinium pusillum* Fresenius |  |
|  | 5/29/19 | *Microcystis* spp. |  |
|  | 6/03/19 | *Microcystis* spp. | *Stephanodiscus hantzschii* Grunow |
|  | 6/11/19 | *Microcystis* spp. |  |
|  | 6/17/19 | *Microcystis* spp. |  |
|  | 6/24/19 | *Microcystis* spp. |  |
|  | 7/01/19 | *Microcystis* spp. |  |
|  | 7/10/19 | *Microcystis* spp. |  |
|  | 7/17/19 | *Microcystis* spp. | *Micractinium pusillum* Fresenius |
|  | 8/09/19 | *Micractinium pusillum* Fresenius |  |
|  | 8/22/19 | *Micractinium pusillum* Fresenius |  |
|  | 9/03/19 | *Stephanodiscus hantzschii* Grunow |  |
|  | 9/17/19 | *Stephanodiscus hantzschii* Grunow |  |
| G3 | 6/03/19 | *Pseudanabaena* cf. *limnetica* (Lemmermann) Komárek |  |
|  | 8/22/19 | *Scenedesmus ecornis* (Ehrenberg) Chodat | *Choricystis* sp. |
|  | 9/03/19 | *Micractinium pusillum* Fresenius |  |
|  | 9/17/19 | *Micractinium pusillum* Fresenius | *Microcystis* spp. |

**Supplementary Table 5.** The results of intrageneric classification using intersecting ranges on another sample. The sample was collected from Mesocosm tank D2 in 09/03/2019. (A) Confusion matrices for used filter sets with precision values for correct predictions (highlighted in green) and false discovery rate for misclassifications (highlighted in orange). (B) Percentage values for accuracy and reliability (precision) of used methods. Overall performance for intergeneric and intrageneric classification in percentage is highlighted in blue.


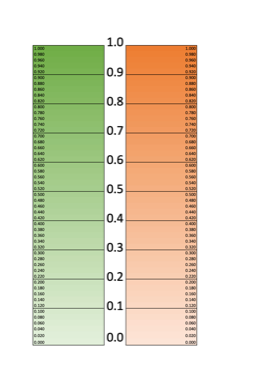

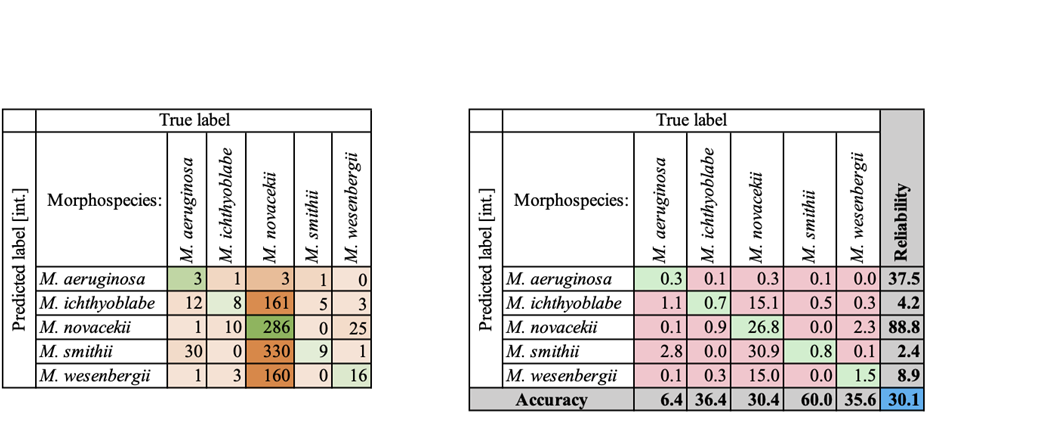


**B**

**A**
